# Elevated Serum IGFBP-2 and CTGF Levels Are Associated with Disease Activity in Patients with Dermatomyositis

**DOI:** 10.1155/2022/9223883

**Published:** 2022-03-20

**Authors:** Min Yang, Yuehong Chen, Geng Yin, Sang Lin, Huan Liu, Yupeng Huang, Yunru Tian, Yueyuan Zhou, Fengming Luo, Qibing Xie

**Affiliations:** ^1^Department of Rheumatology and Immunology, West China Hospital, Sichuan University, Chengdu 610041, China; ^2^Department of Pulmonary and Critical Care Medicine, West China Hospital, Sichuan University, 37 Guo Xue Xiang, Chengdu 610041, China

## Abstract

**Background:**

Insulin-like growth factor-binding proteins (IGFBPs) and connective tissue growth factor (CTGF) participate in angiogenesis. Dermatomyositis (DM) is characterized by microvasculopathy-derived skin lesions. Here, we investigated the clinical significance of serum IGFBP and CTGF levels in DM patients.

**Methods:**

In this study, 65 DM patients and 30 healthy controls were enrolled. Serum IGFBP and CTGF levels were examined by ELISA, and their correlation with clinical and laboratory findings was analyzed by Spearman's correlation.

**Results:**

Serum IGFBP-2, IGFBP-4, and CTGF levels were higher in DM patients than in healthy controls (median (quartile): 258.9 (176.4–326.1) ng/mL vs. 167.7 (116.1–209.4) ng/mL, *p* < 0.0001; 450.4 (327.3–631.8) ng/mL vs. 392.2 (339.0–480.2) ng/mL, *p* = 0.04; and 45.71 (38.54–57.45) ng/mL vs. 35.52 (30.23–41.52) ng/mL, *p* = 0.001, respectively). IGFBP-2 and CTGF levels were positively correlated with cutaneous (*r* = 0.257, *p* = 0.040 and *r* = 0.427, *p* = 0.015, respectively) and global (*r* = 0.380, *p* = 0.002 and *r* = 0.292, *p* = 0.019, respectively) disease activity in DM patients.

**Conclusion:**

Serum IGFBP-2 and CTGF levels were increased in patients with DM and correlated with cutaneous and global disease activity.

## 1. Introduction

Dermatomyositis (DM) is a chronic autoimmune disorder that mostly occurs in adults over 40 years of age and is characterized by skin changes, multiple organ involvement, and presentation of myositis-specific antibodies [1]. The estimated incidence of DM is 9.63 cases per million [2], and its main feature is the presence of skin lesions, which can present as patches, plaques, follicular papules, ichthyosis, ulcerations, and isolated edema. The most typical cutaneous manifestations are Gottron's sign and heliotrope rash [3, 4]. Microvasculopathy plays an important role in the pathology of cutaneous lesions and is displayed as endothelial cell degeneration with separation from the basement membrane, obvious reduction of superficial vascular density, occlusive fibrin thrombi caused by intraluminal fibrin deposition, C_5b-9_ deposition in blood vessels, ablation of dermal papillae capillaries, residual vessels showing ectasia, and hypovascularity [5, 6].

DM diagnosis is based on clinical manifestations such as skin lesions and muscle abnormalities. Nevertheless, serological testing for myositis-specific antibodies has an indispensable role in assisting the diagnosis and differentiation of DM patients [7, 8]. To date, nearly twenty myositis-specific autoantibodies have been reported in the clinic [9]. However, the positive detection of each antibody ranged from 0% to 48%, and all antibodies covered approximately 70% of patients with DM. Thus, more biomarkers correlating with clinical signs and disease activity are needed for the identification of DM patients [9].

Insulin-like growth factor-binding proteins (IGFBPs) consist of six proteins named IGFBP-1−6, and although they share high homology, they have distinct functions. IGFBPs are involved in the regulation of proliferation, survival, invasion, differentiation, migration, and angiogenesis, thus regulating a wide range of physiological and pathological processes, including metabolism, immune regulation, cancer, and neurological diseases [10]. Connective tissue growth factor (CTGF) belongs to the IGFBP superfamily and shares the conserved N-terminal domain of IGFBPs, although it has a lower binding affinity to IGF ligands [11].

IGFBPs are expressed in vascular smooth muscle cells and endothelial cells of blood vessels, and their expression is regulated by many factors, including growth factors, hemodynamic forces, and cytokines. IGFBPs play a role in the pathology of vasculopathy [12]. Since microvasculopathy is involved in the pathology of DM skin lesions, we carried out this novel study to assess the clinical significance of IGFBPs and CTGF in DM patients and found that elevated serum levels of these proteins are associated with myositis disease activity.

## 2. Materials and Methods

### 2.1. Study Population

This is a cross-sectional study that enrolled DM patients with or without interstitial lung disease (ILD) who visited the Department of Rheumatology in West China Hospital of Sichuan University between December 2019 and November 2020. The DM patients were over 18 years of age and were diagnosed according to the Bohan and Peter criteria [13, 14]. Exclusion criteria included the following: other lung diseases including idiopathic pulmonary fibrosis (IPF), pulmonary sarcoidosis, pulmonary infection, and chronic obstructive pulmonary disease; autoimmune diseases other than DM; malignant diseases; pregnancy; and overall poor condition. A total of 32 DM patients without ILD (DM-non-ILD) and 33 DM patients with ILD (DM-ILD) were enrolled. Healthy volunteers, matched with DM by age and gender, were enrolled as healthy controls (HCs) (*n* = 30). The detailed information of DM patients and healthy controls is listed in Supplementary Table 1. ILD was confirmed by high-resolution computed tomography (HRCT). Pulmonary function was evaluated upon enrollment.

### 2.2. Ethics and Consent to Participate

The study complied with the Declaration of Helsinki and was approved by the ethics committee of West China Hospital (No. 246 in 2019), and written informed consent was obtained from all participants. All methods were carried out in accordance with the relevant guidelines and regulations.

### 2.3. Myositis Disease Activity Assessment

Two experienced rheumatologists collected information regarding patients' previous medical history and performed physical examinations during the clinical assessment. Disease activity was measured using the myositis intention to treat activity index (MITAX) from the myositis disease activity assessment tool [15]. The global disease activity score of MITAX is the sum of the worst category scores for each of the seven individual organ systems (constitutional, cutaneous, skeletal, gastrointestinal, pulmonary, cardiac, and muscle) divided by the maximum possible score (range = 1 − 63). For each system, the scores are classified as five categories, A (active), B (beware), C (contentment), D (discount), and E (no evidence), corresponding to 9 points, 3 points, 1 point, 0 points (no current activity but known to have been active in the past), and 0 points (no current or previous activity), respectively. Thus, a high score indicates high disease activity. Disease activity was assessed by the physician at the time of enrollment.

### 2.4. Detection of Serum IGFBP and CTGF Levels

A total of 4 mL venous blood was collected from each patient and HC in a blood collection tube with coagulating agent, and the samples were allowed to clot at room temperature for approximately 2 h. After clot formation, the samples were centrifuged at 2000*g* for 10 min. Then, the sera were collected and stored at −80°C.

Serum IGFBP and CTGF levels were detected using the following human ELISA kits: IGFBP-1 (ab213789, Abcam Cambridge, UK), IGFBP-2 (ab215082, Abcam), IGFBP-3 (EHIGFBP3, Thermo Fisher Scientific, Waltham, MA, USA), IGFBP-4 (ab230936, Abcam), IGFBP-6 (EHIGFBP6, Thermo Fisher Scientific), and CTGF (ab261851, Abcam).

### 2.5. Statistical Analysis

All statistical analyses were conducted using SPSS Statistics 22.0 and GraphPad Prism 6.0. Normal distribution of continuous variables was evaluated by the Kolmogorov-Smirnov test. The data were expressed as the mean ± standard deviation (SD), median (quartile), or number (percentage). The Mann-Whitney *U*-test was used to compare differences between the two groups of continuous variables. Correlations between serum IGFBP and CTGF levels and clinical characteristics, including myositis disease activity, laboratory findings, pulmonary function, and activity and damage of cutaneous manifestations, were analyzed by Spearman's correlation coefficient (*r*). A *p* value of less than 0.05 was considered statistically significant.

## 3. Results

### 3.1. Baseline Characteristics

The baseline characteristics of the patients are summarized in Table 1, and detailed information of all patients and controls is listed in Supplementary Table 1. A total of 65 DM patients with an age range of 19-68 years and 30 healthy controls with an age range of 31-65 years were included. The mean age of the patients was 47.71 years, and 73.8% were female. In contrast, the mean age of the healthy controls was 47.73 years, and 86.7% were female. Approximately half of the patients had concomitant ILD. The percentage of people testing positive for ANA, anti-MDA-5, and anti-Ro-52 antibodies was 53.85%, 41.54%, and 33.85%, respectively. Almost all DM patients (98.46%) received glucocorticoids, and nearly 90% were using more than two immunosuppressors, such as hydroxychloroquine, mycophenolate mofetil, methotrexate, tacrolimus, and cyclophosphamide. For ILD treatment, approximately half of the DM-ILD patients (54.5%) were using Fluimucil, and nearly one-third (36.4%) were taking pirfenidone.

### 3.2. Serum IGFBP and CTGF Levels

**Figure 1:**
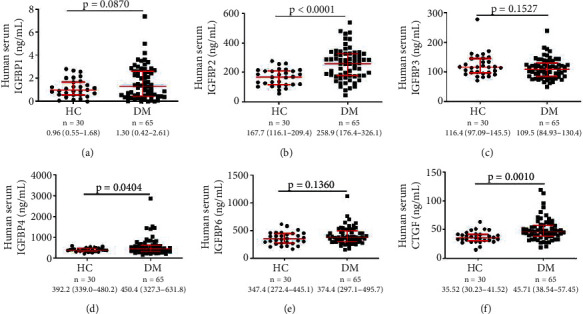
Serum levels of IGFBPs detected by ELISA in HCs and patients with DM. (a) IGFBP-1. (b) IGFBP-2. (c) IGFBP-3. (d) IGFBP-4. (e) IGFBP-6. (f) CTGF. Data are expressed as median (quartile). HC: healthy control; DM: dermatomyositis.

The serum levels of IGFBP-2, IGFBP-4, and CTGF were higher in DM patients than in HCs (258.9 (176.4–326.1) ng/mL vs. 167.7 (116.1–209.4) ng/mL, *p* < 0.0001; 450.4 (327.3–631.8) ng/mL vs. 392.2 (339.0–480.2) ng/mL, *p* = 0.0404; and 45.71 (38.54–57.45) ng/mL vs. 35.52 (30.23–41.52) ng/mL, *p* = 0.001, respectively) (Figures 1(b), 1(d), and 1(f)). In contrast, the serum levels of IGFBP-1, IGFBP-3, and IGFBP-6 in DM patients were comparable to those in HCs (Figures 1(a), 1(c), and 1(e)). Further analysis indicated that the presence of ILD did not influence the serum levels of IGFBP-2, IGFBP-4, and CTGF (Supplementary Figure 1 A-C).

Since anti-MDA5 and anti-Ro-52 antibodies affect the response to treatment and prognosis in patients with DM-ILD, we examined whether there was any difference in IGFBP levels between patients tested positive and those tested negative for these antibodies. There were no differences between the two groups (Supplementary Figure 2 A-F and Supplementary Figure 3 A-F). In addition, since smoking affects the secretory function of epithelial cells [16], we evaluated the effect of smoking on IGFBP and CTGF levels in DM patients and found that the levels were not affected by smoking (Supplementary Figure 4 A-F).

### 3.3. Correlation between IGFBP Levels and Clinical Characteristics

#### 3.3.1. Correlation with Course of Disease

The serum IGFBP-1 level was negatively correlated with the course of DM in all patients (*r* = −0.261, *p* = 0.037) and in patients without ILD (*r* = −0.419, *p* = 0.017). Moreover, CTGF level was negatively correlated with the course of the disease in DM patients without ILD (*r* = −0.397, *p* = 0.024). In contrast, IGFBP-2, IGFBP-3, IGFBP-4, and IGFBP-6 levels did not correlate with the course of DM. None of the IGFBP levels correlated with the course of ILD (Supplemental Table 2).

#### 3.3.2. Correlation with Myositis Disease Activity

IGFBP levels were correlated with disease activity in patients with DM (Table 2). The IGFBP-1 level was positively correlated with constitutional (*r* = 0.472, *p* < 0.001), cutaneous (*r* = 0.270, *p* = 0.031), gastrointestinal (*r* = 0.415, *p* = 0.001), cardiovascular (*r* = 0.367, *p* = 0.030), muscle (*r* = 0.406, *p* = 0.001), and global (*r* = 0.584, *p* < 0.001) disease activity in all DM patients. The IGFBP-2 level was positively correlated with constitutional (*r* = 0.260, *p* = 0.038), cutaneous (*r* = 0.257, *p* = 0.040), gastrointestinal (*r* = 0.330, *p* = 0.008), muscle (*r* = 0.336, *p* = 0.007), and global (*r* = 0.380, *p* = 0.002) disease activity in all DM patients. The IGFBP-3 level was negatively associated with cutaneous disease activity (*r* = −0.346, *p* = 0.0005) in all DM patients. The IGFBP-4 level was correlated with pulmonary (*r* = 0.300, *p* = 0.016), cardiovascular (*r* = 0.263, *p* = 0.036), and global (*r* = 0.280, *p* = 0.025) disease activity in all DM patients. The CTGF level was positively correlated with constitutional (*r* = 0.247, *p* = 0.049), cutaneous (*r* = 0.427, *p* = 0.015), and global (*r* = 0.292, *p* = 0.019) disease activity in patients with DM. In contrast, the IGFBP-6 level was not correlated with disease activity in any organ.

#### 3.3.3. Correlation with Pulmonary Function

The correlation of IGFBP levels with pulmonary function in DM-ILD patients was assessed by Spearman's correlation (Supplementary Table 3). Both IGFBP-2 and CTGF levels were positively correlated with dyspnea (*r* = 0.392, *p* = 0.027 and *r* = 0.45, *p* = 0.01, respectively), while IGFBP-4 levels were correlated with predicted forced vital capacity (*r* = 0.47, *p* = 0.026), predicted forced expiratory volume in one second (*r* = 0.46, *p* = 0.033), total lung capacity (TLC) (*r* = 0.455, *p* = 0.034), and predicted TLC (*r* = 0.499, *p* = 0.018).

### 3.4. Correlation with Cutaneous Manifestations

Since skin lesions are one of the major presentations in patients with DM and IGFBPs can modulate angiogenesis, we examined the associations of IGFBPs serum levels with skin disease activity and damage which were assessed by cutaneous assessment tool (CAT). IGFBP-1, IGFBP-2, and CTGF levels were positively correlated with skin disease activity (*r* = 0.588, *p* < 0.001, *r* = 0.396, *p* = 0.001, and *r* = 0.325, *p* = 0.009, respectively) and skin disease damage (*r* = 0.551, *p* < 0.001, *r* = 0.348, *p* = 0.005, and *r* = 0.283, *p* = 0.024, respectively), while IGFBP-3 levels were negatively associated with skin disease activity (*r* = −0.294, *p* = 0.018) and skin disease damage (*r* = −0.324, *p* = 0.009) (Table 3).

## 4. Discussion

Dermatomyositis (DM) is a chronic autoimmune disorder characterized by microvasculopathy-derived skin lesions. Serological testing for myositis-specific antibodies has an indispensable role in assisting the diagnosis and differentiation of DM patients. However, serum diagnostic biomarkers are not sufficient as they do not accurately diagnose all patients. Therefore, novel biological markers for DM diagnosis are needed. IGBFPs and CTGF are involved in physiological and pathological processes of angiogenesis. Thus, we hypothesized that they play a role in DM through microvasculopathy. To examine this hypothesis, we investigated the clinical significance and diagnostic value of serum IGBFP and CTGF levels in DM patients for the first time. We found that IGFBP-2, IGFBP-4, and CTGF levels were higher in DM patients than in HCs. Serum IGFBP-1, IGFBP-2, and CTGF levels were positively correlated with cutaneous and global disease activity and differed among the patients according to the severity of skin lesions and global disease. IGFBP-4 levels were correlated with pulmonary disease activity and several lung function indicators.

Previous studies have reported that IGFBPs play critical roles in physiological and pathological processes of angiogenesis. While IGFBP-2 has a proangiogenic role, IGFBP4–6 have antiangiogenic effects. In contrast, IGFBP-1 and IGFBP-3 have shown both proangiogenic and antiangiogenic roles under different contexts [17]. CTGF belongs to the CCN family, which plays various roles in angiogenesis and tumor growth. CTGF induces angiogenesis in endothelial cells by promoting tube formation and plays a role in cancer [18]. DM is a condition characterized by microvascular lesions, and our results demonstrated that IGFBP2 and CTGF levels are associated with cutaneous disease activity. Therefore, we concluded that IGFBPs are involved in the pathology of DM. However, further investigation is required to understand the underlying mechanisms whereby IGFBPs regulate the pathology of cutaneous lesions.

IGFBPs can be secreted by epithelial cells in bronchioles and alveoli, endothelial cells, fibroblasts, and myofibroblasts and are present in bronchoalveolar lavage (BAL) fluid and serum [11]. Recent studies have reported elevated levels of IGFBPs, including IGFBP2–5 and CTGF, in lung samples, including BAL cells, BAL fluids, and lung fibroblasts of patients with idiopathic IPF, suggesting that IGFBPs might play a profibrogenic role in this disorder [11, 19–23]. Specifically, they were shown to increase the production of extracellular matrix (ECM) by primary lung fibroblasts and myofibroblasts transdifferentiated from fibroblasts or epithelial cells, while reducing the degradation of ECM. In addition, they were shown to participate in the activation and transdifferentiation of fibroblasts as well as in epithelial-mesenchymal transition from epithelial cells. To be specific, IGFBP-3 and IGFBP-5 overexpression could increase the production of matrix fibronectin 14-fold and 16-fold, respectively, while overexpression of IGFBP-5 led to the increased secretion and deposition of fibronectin and collagen type I in the ECM of fibroblasts [24]. CTGF stimulates the production of ECM by fibroblasts, whereas the inhibition of CTGF activity by neutralizing antibodies or suppression of gene expression significantly lowers TGF*β*-stimulated collagen production [25, 26]. In addition, ECM components can promote the production of IGFBPs, thus constituting a feedback mechanism for the regulation of IGFBP activity. Furthermore, binding of IGFBPs to ECM provides a stable environment that protects them from proteolytic degradation [27].

Since IGFBPs play roles in lung fibrosis and 10–45% of patients with DM have pulmonary involvement presenting as ILD [28], we assessed the differences in IGFBP serum levels between DM patients with and without ILD; however, no differences were observed. This may be explained by the fact that in patients with chronic IPF, the most important histological pattern of ILD is usual interstitial pneumonia (UIP), while in DM patients, the major histological forms of ILD are nonspecific interstitial pneumonia and organizing pneumonia [29, 30]. Since serum IGFBP-2 levels are increased in IPF, it might be related to UIP. Although we assume that IGFBPs are involved in different histological forms of ILD, we could not perform further analysis of serum IGFBP levels in DM-ILD patients with different histological types owing to the limited number of patients. IPF is the major type of ILD, accounting for about 25% of all ILD cases [31]. Lung fibrosis is initiated in response to injury and under certain disease conditions in which autoantibodies, genetics, and innate and adaptive immunity play different roles, which might explain the different forms of lung fibrosis and inflammation [30].

A previous study suggested that IGFBP-4 does not have a fibrotic role in dermal fibrosis and thickening using a human skin organ culture model [32]. Another study found that IGFBP-4 has an antifibrotic role in systemic sclerosis, a prototypic fibrotic disorder [33], through reducing TGF*β*-induced ECM production and the expression of the profibrotic factor CTGF [33]. Although we found that IGFBP-4 was correlated with pulmonary disease activity and lung function, serum IGFBP-4 levels did not differ among DM patients with and without ILD. Thus, its possible role in fibrosis requires further investigation.

IGFBP-3 has been associated with different cancers, showing both promoting and inhibiting actions. The serum levels of IGFBP-3 in patients with melanoma are significantly reduced, and a low IGFBP-3 level is associated with metastasis and cancer progression, while IGFBP-3 administration can significantly inhibit the migration and invasion of different melanoma cell lines [34, 35]. Moreover, high IGFBP-3 levels may have a protective effect against breast cancer [36]. However, IGFBP-3 overexpression has been associated with the progression and metastasis of several cancer types, including cutaneous squamous cell carcinoma and nasopharyngeal carcinoma [37, 38]. In our study, IGFBP-3 levels were not correlated with disease activity, other laboratory findings, or lung function in DM patients. Approximately 15% of adult DM patients are estimated to have cancer [39]. Owing to the limited number of patients in our study, and the fact that we did not focus on the subgroup of DM patients with cancer, we did not perform further analysis. Thus, further studies are required to elucidate whether IGFBP-3 plays a role in DM patients with cancer.

There are several limitations in our study. First, a previous study reported that IGFBP-2 is significantly increased in patients with IPF compared with HCs and that the level of IGFBP-2 in the patients is significantly reduced after antifibrotic treatment with pirfenidone and nintedanib [19]. However, our study did not examine differences in IGFBP levels before and after antifibrotic treatment. Second, the limited number of study subjects did not allow us to examine differences in IGFBP levels among different subgroups of DM patients with regard to different additional pathologies, such as cancer. Third, our study was only powered for the analysis of IGFBP2 and CTGF. Therefore, the lack of statistical significance in some results might be related to the small sample size.

## 5. Conclusions

Serum IGFBP-2 and CTGF levels may potentially be used to assess the severity of skin lesions and global disease in DM. Serum IGFBP-4 levels may potentially be used to assess lung function in DM-ILD patients. However, further studies with larger sample sizes are needed to confirm these results.

## Figures and Tables

**Table 1:** Characteristics of patients with DM.

Characteristics	HC (*n* = 30)	DM (*n* = 65)
Age (years, mean ± SD)	47.73 ± 7.64	47.71 ± 10.88
Gender (F/M)	26/4	48/17
ILD (Y/N)		33/32
ANA (+)		35 (53.85)
Anti-MDA-5 (+)		27 (41.54)
Anti-Ro-52 (+)		22 (33.85)
Treatments for DM		
Glucocorticoids		64 (98.46)
More than two immunosuppressors		59 (90.77)
Treatments for ILD		
Pirfenidone		12 (36.4)
Fluimucil		18 (54.5)
CRP (mg/L, median and quartile)		3.11 (1.43, 5.77)
CK (IU/L, median and quartile)		60 (39, 133)
LDH (IU/L, median and quartile)		247 (215, 343)
HBDH (IU/L, median and quartile)		194 (173, 285)

**Table 2:** Spearman correlations of IGFBPs with disease activity assessed by MITAX.

			IGFBP1		IGFBP2		IGFBP3		IGFBP4		IGFBP6		CTGF	
Characteristics	Subgroup patients	Sample size	*r*	*p* value	*r*	*p* value	*r*	*p* value	*r*	*p* value	*r*	*p* value	*r*	*p* value
Constitutional	All DM patients	64	**0.472**	**<0.001**	**0.260**	**0.038**	-0.078	0.540	0.226	0.072	-0.010	0.939	**0.247**	**0.049**
	DM-non-ILD	32	**0.446**	**0.011**	0.326	0.069	-0.191	0.295	0.302	0.093	-0.017	0.926	0.299	0.097
	DM-ILD	32	**0.508**	**0.003**	0.168	0.357	0.059	0.750	0.123	0.502	-0.016	0.929	0.180	0.325
Cutaneous disease activity	All DM patients	64	**0.270**	**0.031**	**0.257**	**0.040**	**-0.346**	**0.005**	0.026	0.837	0.063	0.622	0.233	0.064
	DM-non-ILD	32	0.227	0.211	0.108	0.557	-0.161	0.377	0.265	0.142	-0.010	0.958	**0.427**	**0.015**
	DM-ILD	32	**0.365**	**0.040**	**0.436**	**0.013**	**-0.471**	**0.007**	-0.157	0.392	0.158	0.387	0.070	0.704
Skeletal disease activity	All DM patients	64	0.054	0.672	-1.330	0.293	0.142	0.264	0.001	0.992	-0.136	0.284	0.011	0.929
	DM-non-ILD	32	0.134	0.463	-0.093	0.612	0.059	0.748	0.013	0.946	-0.083	0.650	-0.003	0.986
	DM-ILD	32	-0.052	0.779	-0.190	0.299	0.190	0.299	0.010	0.955	-0.176	0.336	-0.017	0.925
Gastrointestinal disease activity	All DM patients	64	**0.415**	**0.001**	**0.330**	**0.008**	-0.104	0.411	0.188	0.136	-0.067	0.598	0.134	0.290
	DM-non-ILD	32	**0.385**	**0.030**	0.160	0.382	0.003	0.987	0.264	0.144	-0.276	0.126	0.220	0.226
	DM-ILD	32	**0.461**	**0.008**	**0.479**	**0.006**	-0.151	0.410	0.170	0.352	0.161	0.377	0.038	0.838
Pulmonary disease activity	All DM patients	64	0.169	0.181	0.135	0.287	0.105	0.407	**0.300**	**0.016**	0.080	0.531	0.049	0.698
	DM-non-ILD	32	0.254	0.161	-0.184	0.314	0.070	0.704	0.212	0.244	-0.108	0.556	0.129	0.483
	DM-ILD	32	**0.420**	**0.017**	0.338	0.058	-0.046	0.803	**0.391**	**0.027**	0.061	0.742	0.130	0.480
Cardiovascular disease activity	All DM patients	64	**0.367**	**0.030**	0.184	0.145	-0.071	0.578	**0.263**	**0.036**	0.052	0.683	0.229	0.069
	DM-non-ILD	32	**0.517**	**0.002**	0.331	0.064	-0.079	0.666	**0.4050.405**	**0.021**	0.126	0.493	0.322	0.073
Muscle disease activity	All DM patients	64	**0.406**	**0.001**	**0.336**	**0.007**	-0.087	0.493	0.094	0.458	-0.079	0.536	0.192	0.128
	DM-non-ILD	32	**0.496**	**0.004**	0.249	0.169	-0.161	0.379	0.174	0.342	-0.038	0.837	0.220	0.226
	DM-ILD	32	**0.382**	**0.031**	**0.481**	**0.005**	0.075	0.684	0.225	0.216	0.014	0.940	0.191	0.294
Global disease activity	All DM patients	64	**0.584**	**< 0.001**	**0.380**	**0.002**	-0.183	0.147	**0.280**	**0.025**	0.014	0.915	**0.292**	**0.019**
	DM-non-ILD	32	**0.589**	**< 0.001**	0.261	0.150	-0.164	0.368	**0.386**	**0.029**	-0.053	0.772	**0.394**	**0.026**
	DM-ILD	32	**0.629**	**< 0.001**	**0.478**	**0.006**	-0.203	0.266	0.176	0.336	0.077	0.674	0.113	0.537

Bold fonts indicate statistical significance.

**Table 3:** Spearman correlations of IGFBPs with cutaneous manifestations assessed by cutaneous assessment tool.

Characteristics	Sample size	IGFBP1		IGFBP2		IGFBP3		IGFBP4		IGFBP6		CTGF	
		*r*	*p* value	*r*	*p* value	*r*	*p* value	*r*	*p* value	*r*	*p* value	*r*	*p* value
Skin disease activity	64	**0.588**	**<0.001**	**0.396**	**0.001**	**-0.294**	**0.018**	0.232	0.065	0.240	0.056	**0.325**	**0.009**
Skin disease damage	64	**0.551**	**<0.001**	**0.348**	**0.005**	**-0.324**	**0.009**	0.178	0.159	0.219	0.082	**0.283**	**0.024**

Bold fonts indicate statistical significance.

## Data Availability

The data supporting the conclusions of this article are included within the article and its additional file.

## Supplementary Materials

Supplementary Materials The supplementary document contains four supplemental figures and three supplemental tables. Supplemental Figure 1: serum level of IGFBPs detected by ELISA in DM patients with or without ILD. (A) IGFBP-2. (B) IGFBP-4. (C) CTGF. Data are expressed as median (quartile). Supplemental Figure 2: influence of positive detection of MDA5 on the serum levels of IGFBPs detected by ELISA in patients with DM. (A) IGFBP-1. (B) IGFBP-2. (C) IGFBP-3. (D) IGFBP-4. (E) IGFBP-6. (F) CTGF. Data are expressed as median (quartile). Supplemental Figure 3: effect of positive detection of Ro-52 on serum levels of IGFBPs detected by ELISA in DM patients. (A) IGFBP-1. (B) IGFBP-2. (C) IGFBP-3. (D) IGFBP-4. (E) IGFBP-6. (F) CTGF. Data are expressed as median (quartile). Supplemental Figure 4: effect of smoking on serum levels of IGFBPs detected by ELISA in patients with DM. (A) IGFBP-1. (B) IGFBP-2. (C) IGFBP-3. (D) IGFBP-4. (E) IGFBP-6. (F) CTGF. Data are expressed as median (quartile). Supplemental Table 1: detailed information of enrolled participants. Supplemental Table 2: the Spearman correlations of IGFBPs with the course of disease. Supplemental Table 3: the Spearman correlations of IGFBPs with pulmonary function in patients with DM-ILD.
