# Supplementary material for: Elevated Serum IGFBP-2 and CTGF Levels Are Associated with Disease Activity in Patients with Dermatomyositis
Source: Dis Markers. 2022 Mar 20;2022:9223883. doi: 10.1155/2022/9223883 (PMC8958080; doi:10.1155/2022/9223883)
Supplement: Supplementary Materials — The supplementary document contains four supplemental figures and three supplemental tables. Supplemental Figure 1: serum level of IGFBPs detected by ELISA in DM patients with or without ILD. (A) IGFBP-2. (B) IGFBP-4. (C) CTGF. Data are expressed as median (quartile). Supplemental Figure 2: influence of positive detection of MDA5 on the serum levels of IGFBPs detected by ELISA in patients with DM. (A) IGFBP-1. (B) IGFBP-2. (C) IGFBP-3. (D) IGFBP-4. (E) IGFBP-6. (F) CTGF. Data are expressed as median (quartile). Supplemental Figure 3: effect of positive detection of Ro-52 on serum levels of IGFBPs detected by ELISA in DM patients. (A) IGFBP-1. (B) IGFBP-2. (C) IGFBP-3. (D) IGFBP-4. (E) IGFBP-6. (F) CTGF. Data are expressed as median (quartile). Supplemental Figure 4: effect of smoking on serum levels of IGFBPs detected by ELISA in patients with DM. (A) IGFBP-1. (B) IGFBP-2. (C) IGFBP-3. (D) IGFBP-4. (E) IGFBP-6. (F) CTGF. Data are expressed as median (quartile). Supplemental Table 1: detailed information of enrolled participants. Supplemental Table 2: the Spearman correlations of IGFBPs with the course of disease. Supplemental Table 3: the Spearman correlations of IGFBPs with pulmonary function in patients with DM-ILD. [file 9223883.f1.docx]

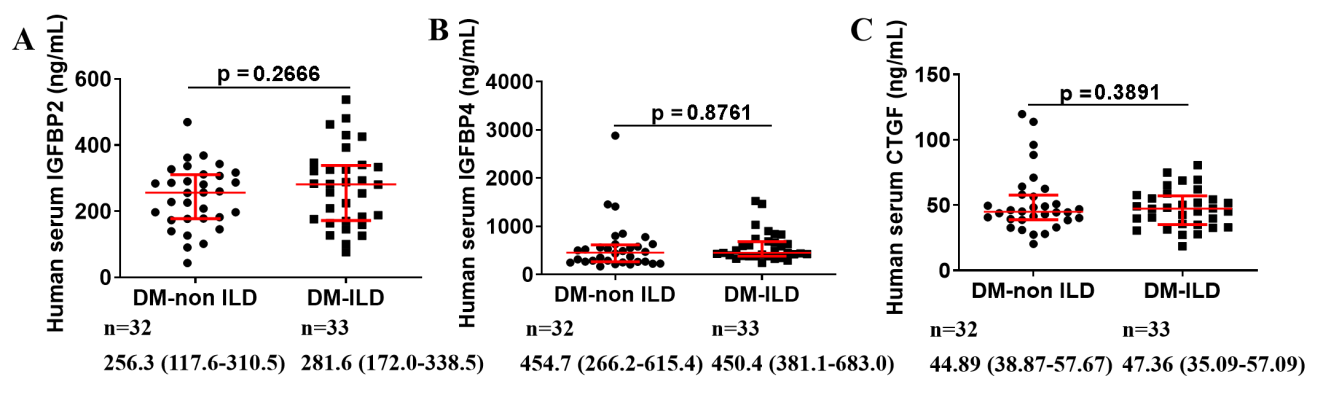


Supplemental Figure 1 Serum level of IGFBPs detected by ELISA in DM patients with or without ILD. (A) IGFBP-2. (B) IGFBP-4. (C) CTGF. Data are expressed as median (quartile).


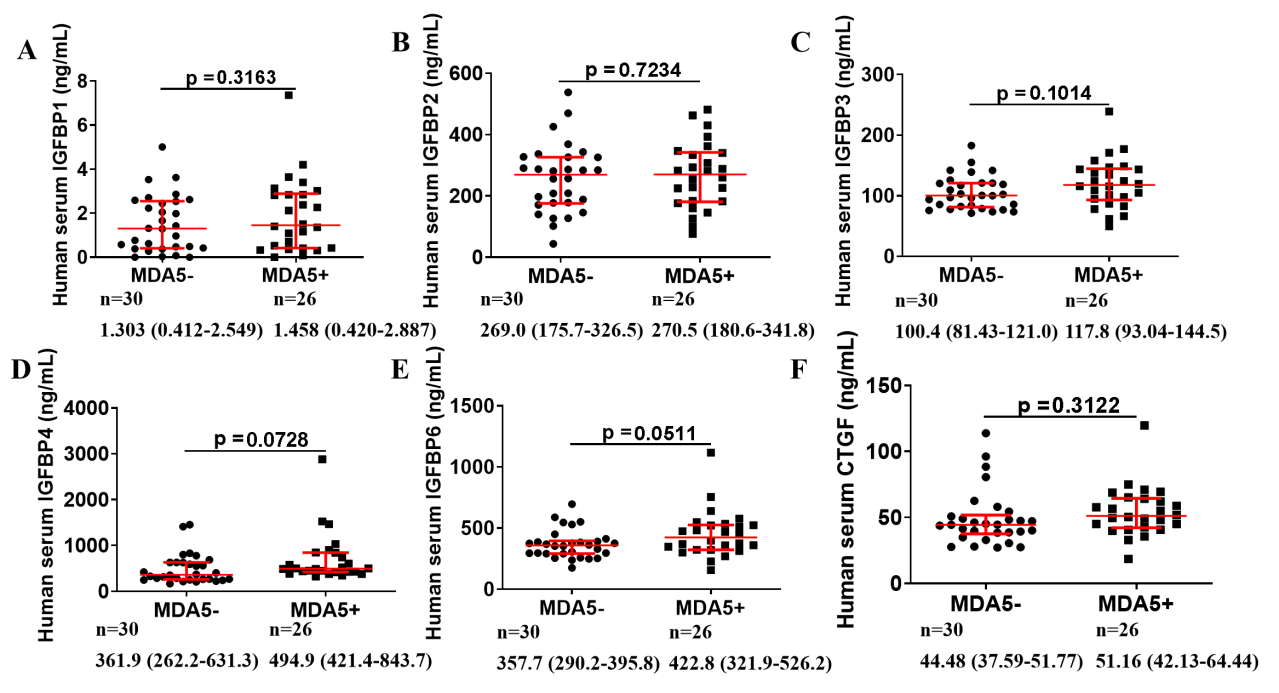


Supplemental Figure 2 Influence of positive detection of MDA5 on the serum levels of IGFBPs detected by ELISA in patients with DM. (A) IGFBP-1. (B) IGFBP-2. (C) IGFBP-3. (D) IGFBP-4. (E) IGFBP-6. (F) CTGF. Data are expressed as median (quartile).


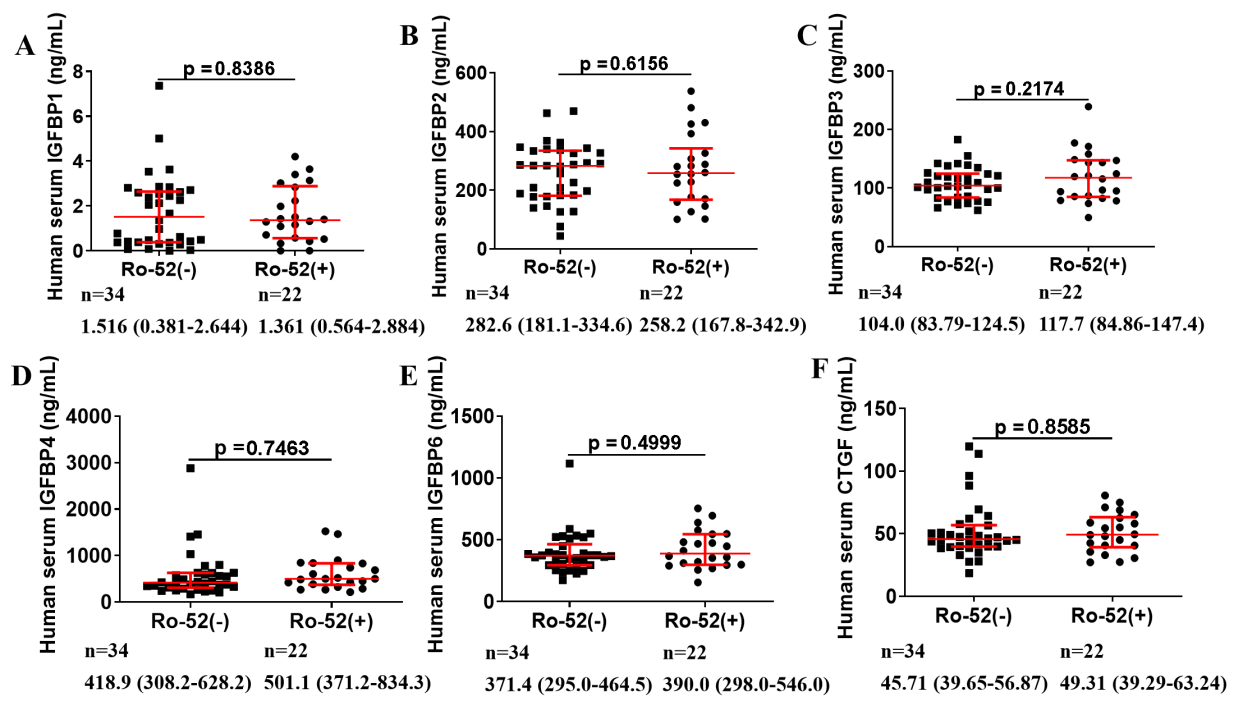


Supplemental Figure 3 Effect of positive detection of Ro-52 on serum levels of IGFBPs detected by ELISA in DM patients. (A) IGFBP-1. (B) IGFBP-2. (C) IGFBP-3. (D) IGFBP-4. (E) IGFBP-6. (F) CTGF. Data are expressed as median (quartile).


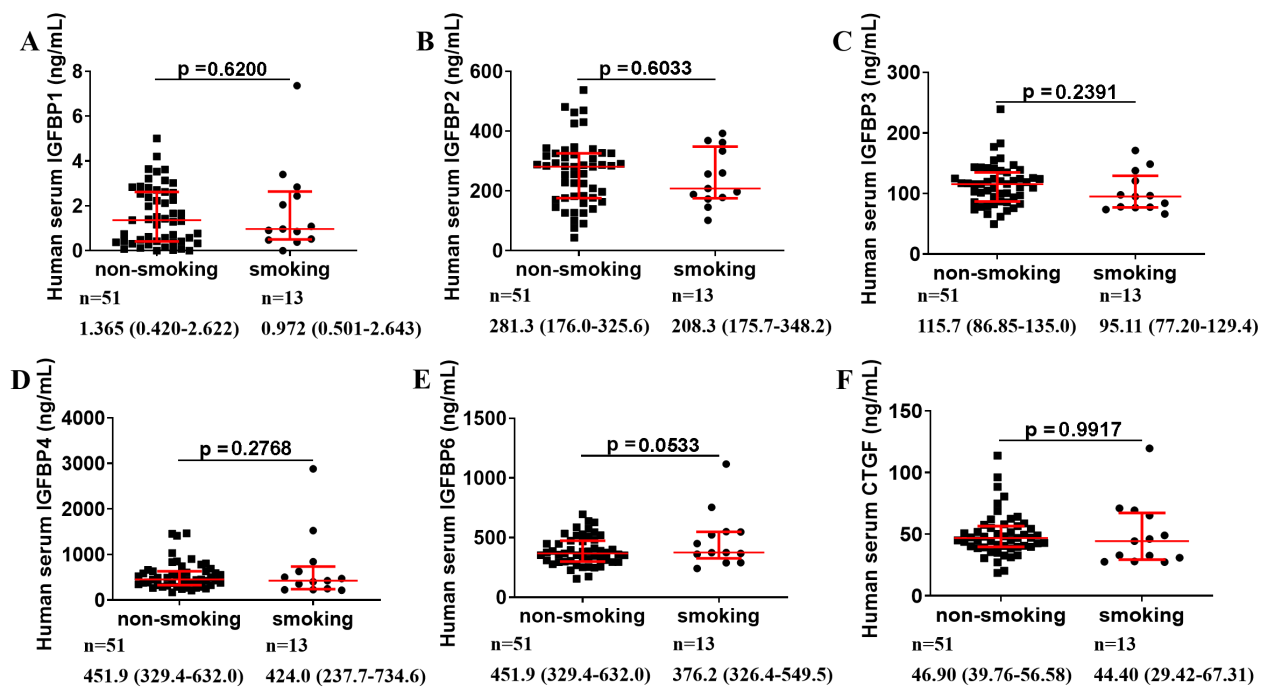


Supplemental Figure 4 Effect of smoking on serum levels of IGFBPs detected by ELISA in patients with DM. (A) IGFBP-1. (B) IGFBP-2. (C) IGFBP-3. (D) IGFBP-4. (E) IGFBP-6. (F) CTGF. Data are expressed as median (quartile).

Supplemental Table 1 Detailed information of enrolled participants

| **NO.** | **Gender** | **Age (years)** | **Height (cm)** | **Body weight (Kg)** | **Smoking** | | **Medical history** | | **Sampling date** | **DM diagnostic date** | | **Treatment for DM** | | **ILD diagnostic date** | | **Treatment for ILD** | | **MSA** | |  |  |  |
| --- | --- | --- | --- | --- | --- | --- | --- | --- | --- | --- | --- | --- | --- | --- | --- | --- | --- | --- | --- | --- | --- | --- |
|  |  |  |  |  |  |  |  |  |  |  |  |  |  |  |  |  |  |  |  |  |  |  |
| DM-ILD* | | | | | | | | | | | | | | | | | | | |  |  |  |
| D1 | F | 43 | 160 | 40 | NO | | NO | | Dec 2019 | May 2019 | | GC, HCQ, TNFi | | Dec 2019 | | GC | | Ro-52(+++) | |  |  |  |
| D2 | F | 52 | 158 | 60 | NO | | Hypothyroidism | | Jan 2020 | July 2017 | | GC, HCQ, CsA | | July 2017 | | Fluimucil | | no detection | |  |  |  |
| D3 | F | 34 | 156 | 51 | NO | | NO | | Jan 2020 | May 2019 | | GC, HCQ, MMF | | May 2019 | | Fluimucil | | MDA-5(++), SRP(+),EJ（+） | |  |  |  |
| D5 | F | 62 | 158 | 65 | NO | | NO | | Jan 2020 | June 2014 | | GC, CTX, gamma globulin | | June 2014 | | Fluimucil | | PL-7(+++) | |  |  |  |
| D11 | F | 54 | 160 | 50 | NO | | T2DM | | Jan 2020 | Dec 2019 | | GC, CTX, HCQ, gamma globulin | | Dec 2019 | | Fluimucil, pirfenidone | | MDA-5(+++), Ro-52(+++) | |  |  |  |
| D12 | F | 49 | 155 | 45 | NO | | NO | | Jan 2020 | Mar 2014 | | GC, MTX, CTX, HCQ, Tripterygium Glycosides | | Mar 2014 | | No treatment | | no detection | |  |  |  |
| D14 | M | 49 | 168 | 66.5 | NO | | Hypertension | | Jan 2020 | Feb 2017 | | GC, TAC, AZA, CTX, HCQ | | Feb 2017 | | Fluimucil, pirfenidone | | MDA-5(+++), Ro-52(+++) | |  |  |  |
| D15 | M | 46 | 168 | 70 | Yes | | NO | | Jan 2020 | Oct 2019 | | GC, MMF | | Oct 2019 | | Tofatib | | MDA-5(++), PM-ScL75(++) | |  |  |  |
| D17 | M | 42 | 162 | 63 | NO | | NO | | Jan 2020 | Dec 2019 | | GC, MTX | | Dec 2019 | | No treatment | | Ro-52(+++) | |  |  |  |
| D18 | F | 43 | 153 | 54.5 | NO | | NO | | Jan 2020 | July 2019 | | GC, CTX, HCQ | | July 2019 | | Fluimucil,tofatib | | MDA-5(+++),Jo-1（+） | |  |  |  |
| D19 | F | 55 | 158 | 56 | NO | | NO | | Jan 2020 | July 2018 | | GC, HCQ, Tripterygium Glycosides | | Oct 2018 | | Fluimucil | | MDA5(++) | |  |  |  |
| D20 | F | 65 | 160 | 60 | NO | | Hypertension | | Dec 2019 | Sep 2019 | | GC, CTX | | Sep 2019 | | Pirfenidone | | MDA-5(+++), Ro-52(+++) | |  |  |  |
| D22 | F | 63 | 154 | 47 | NO | | T2DM | | Dec 2019 | Oct 1999 | | GC, CTX, HCQ, thalidomide | | June 2019 | | CTX | | MDA-5(+) | |  |  |  |
| D23 | F | 43 | 156 | 49 | NO | | NO | | Jan 2020 | May 2019 | | GC, CTX, HCQ, thalidomide | | May 2019 | | No treatment | | MDA-5(+++), Ro-52(+++) | |  |  |  |
| D24 | M | 55 | 165 | 57.5 | NO | | hypothyroidism | | Jan 2020 | Jan 2020 | | GC, CTX, HCQ, gamma globulin | | Jan 2020 | | Fluimucil | | MDA-5(+++), Ro-52(+++) | |  |  |  |
| D25 | F | 56 | 150 | 47 | NO | | Pulmonary hypertension | | Jan 2020 | June 2019 | | GC, TAC | | June 2019 | | Pirfenidone | | MDA-5(++), Ro-52(+++), EJ(+++), NXP（+） | |  |  |  |
| D27 | F | 42 | 156 | 60 | NO | | NO | | Dec 2019 | April 2019- | | GC | | April 2019 | | Fluimucil | | T1F1-r（+） | |  |  |  |
| D28 | M | 48 | 163 | 55 | Yes | | NO | | Dec 2019 | Mar 2019 | | GC, CTX, HCQ | | April 2019 | | pirfenidone, Fluimucil | | MDA-5(++), Ro-52(+++) | |  |  |  |
| D29 | F | 47 | 160 | 50 | NO | | NO | | Dec 2019 | May 2016 | | GC, HCQ | | May 2016 | | No treatment | | MDA-5(+++),Ro-52(+++) | |  |  |  |
| D31 | F | 44 | 151 | 46.5 | NO | | NO | | June 2020 | June 2020 | | GC, TAC | | April 2020 | | Fluimucil, pirfenidone | | PL-7(+), Ro-52 (+) | |  |  |  |
| D32 | F | 53 | 159 | 51 | NO | | NO | | July 2020 | June 2020 | | GC, TAC | | June 2020 | | pirfenidone,Rituximab, | | MDA5(++), Ro-52(+) | |  |  |  |
| D33 | M | 50 | 171 | 86 | NO | | NO | | July 2020 | Nov 2019 | | GC, HCQ | | Sep 2019 | | Fluimucil, CTX | | MDA5 (+) | |  |  |  |
| D34 | F | 50 | 161 | 52 | NO | | NO | | July 2020 | July 2019 | | GC, MMF | | July 2019 | | Fluimucil, CTX | | MDA5 (+), Ro-52 (+) | |  |  |  |
| D35 | F | 45 | 160 | 55 | NO | | NO | | July 2020 | July 2020 | | GC, HCQ, thalidomide | | July 2020 | | GC, CTX | | T1F1-r(++), Ro-52(+++) | |  |  |  |
| D36 | F | 33 | 163 | 49 | NO | | NO | | Aug 2020 | June 2019 | | GC, HCQ, CsA, thalidomide | | July 2019 | | pirfenidone, CTX, Tofacitinib, gamma globulin | | MDA5(+++), nRNP/Sm(+) | |  |  |  |
| D37 | F | 47 | 150 | 50 | NO | | NO | | July 2020 | May 2019 | | GC, TAC, HCQ | | Jan 2012 | | pirfenidone | | PL-12(+++), MDA-5(+), Ro-52(+++), SAE1/SAE2(+), | |  |  |  |
| D38 | F | 57 | 156 | 55 | NO | | Hashimoto thyroiditis | | Aug 2020 | Jan 2019 | | GC, HCQ | | May 2019 | | Fluimucil, CTX | | MDA5(+++), Ro-52(+++), SAE1/SAE2(+) | |  |  |  |
| D39 | F | 44 | 155 | 46 | NO | | Goiter | | July 2020 | Jan 1993 | | GC, MTX | | Jan 2018 | | Fluimucil, CTX | | no detection | |  |  |  |
| D40 | F | 54 | 156 | 54 | NO | | NO | | Aug 2020 | Aug 202 | | GC, CTX | | Aug 2020 | | Fluimucil, pirfenidone | | negative | |  |  |  |
| D41 | M | 68 | 167 | 65 | Yes | | NO | | Aug 2020 | Aug 2020 | | GC, TAC | | Aug 2020 | | pirfenidone | | PL-7(+++) | |  |  |  |
| D42 | F | 28 | 162 | 56 | NO | | NO | | Aug 2020 | June 2020 | | GC, TAC | | June 2020 | | Fluimucil, gamma globulin | | MDA5(+), cN1A(+) | |  |  |  |
| D43 | F | 49 | 150 | 55 | NO | | T2DM, hypertension | | Sep 2020 | Jan 2010 | | GC, HCQ, MTX | | Sep 2020 | | GC | | SRP(+++) | |  |  |  |
| D65 | F | 54 | 158 | 60 | NO | | T2DM, hypertension | | Jan 2020 | Jan 2019 | | GC, TAC, CsA, MTX, gamma globulin | | Jan 2019 | | Fluimucil, pirfenidone | | MDA-5(+++), Ro-52(++) | |  |  |  |
| DM-non-ILD | | | | | | | | | | | | | | | | | | | |  |  |  |
| D4 | F | 66 | 155 | 46 | NO | | T2DM | | Jan 2020 | Dec 2014 | | GC, CTX | | / | | / | | no detection | |  |  |  |
| D6 | M | 38 | 165 | 65 | Yes | | NO | | Jan 2020 | Sep 2019 | | GC, MTX, HCQ | | / | | / | | PM-SCL100（+） | |  |  |  |
| D7 | M | 42 | 170 | 56 | Yes | | NO | | Jan 2020 | Jan 2010 | | GC, MTX, HCQ | | / | | / | | no detection | |  |  |  |
| D8 | F | 51 | 160 | 55 | NO | | NO | | Jan 2020 | June 2019 | | GC, MTX, HCQ, thalidomide | | / | | / | | Mi-2（++） | |  |  |  |
| D9 | M | 47 | 165 | 47 | Yes | | NO | | Jan 2020 | Jan 2020 | | GC | | / | | / | | MDA5(++) | |  |  |  |
| D10 | M | 42 | 173 | 58 | NO | | NO | | Jan 2020 | Feb 2013 | | GC, MTX, HCQ | | / | | / | | no detection | |  |  |  |
| D13 | F | 64 | 155 | 60 | NO | | NO | | Jan 2020 | Jan 1997 | | / | | / | | / | | negative | |  |  |  |
| D16 | F | 38 | 155 | 55 | NO | | NO | | Jan 2020 | May 2019 | | GC, MMF, HCQ | | / | | / | | SAE1/SAE2（+++） | |  |  |  |
| D21 | F | 66 | 155 | 55 | NO | | NO | | Dec 2019 | Dec 2015 | | GC, CTX, HCQ | | / | | / | | MDA-5(++) | |  |  |  |
| D26 | F | 49 | 157 | 65 | NO | | NO | | Dec 2019 | May 2019 | | GC, thalidomide | | / | | / | | PM-Scl75(+) | |  |  |  |
| D30 | F | 42 | 155 | 41.5 | NO | | NO | | Jan 2020 | Jan 2019 | | GC, thalidomide, HCQ | | / | | / | | negative | |  |  |  |
| D44 | F | 58 | 155 | 60 | NO | | Hypertension, hyperglycemia | | June 2020 | May 2020 | | GC, HCQ, CTX | | / | | / | | Mi-2B(+) | |  |  |  |
| D45 | F | 61 | 157 | 52.5 | NO | | Hypertension | | July 2020 | Nov 2019 | | GC, TAC | | / | | / | | Ku (+) | |  |  |  |
| D46 | F | 31 | 155 | 52.5 | NO | | NO | | Aug 2020 | April 2020 | | GC, HCQ, CTX | | / | | / | | nRNP/Sm(+++), Ro-52(+), NXP-2（+） | |  |  |  |
| D47 | M | 57 | 160 | 72 | Yes | | NO | | Aug 2020 | Aug 2020 | | GC, TAC | | / | | / | | NXP-2 (+) | |  |  |  |
| D48 | M | 49 | 170 | 59 | Yes | | Hypothyroidism, hypertriglyceridemia | | Aug 2020 | Aug 2020 | | GC, HCQ, CTX, gamma globulin | | / | | / | | MDA-5(+++), Ro-52(+++) | |  |  |  |
| D49 | M | 52 | 165 | 53 | Yes | | Hypothyroidism, hypertension | | Aug 2020 | Aug 2020 | | GC, MTX | | / | | / | | SRP(+),HMGCR(++) | |  |  |  |
| D50 | M | 25 | 168 | 54 | Yes | | fasciitis | | Sep 2020 | Aug 2020 | | GC, HCQ, thalidomide | | / | | / | | negative | |  |  |  |
| D51 | F | 35 | 150 | 47 | NO | | NO | | Sep 2020 | Sep 2020 | | GC, HCQ, MTX, Tofacitinib | | / | | / | | Mi-2(++),MDA-5 (++), Ro-52(+) | |  |  |  |
| D52 | F | 47 | 154 | 47 | NO | | hyperlipidaemia, T2DM | | Sep 2020 | Aug 2020 | | GC, TAC | | / | | / | | MDA-5(+), Ro-52(++) | |  |  |  |
| D53 | M | 51 | 178 | 60 | Yes | | NO | | Sep 2020 | May 2020 | | GC, HCQ, MTX | | / | | / | | no detection | |  |  |  |
| D54 | F | 42 | 155 | 43 | NO | | Hepatitis B | | Sep 2020 | Jan 2019 | | GC, HCQ, MTX, thalidomide | | / | | / | | negative | |  |  |  |
| D55 | F | 46 | 160 | 70 | Yes | | Hepatitis B | | Sep 2020 | July 2018 | | GC, MTX, HCQ, CsA | | / | | / | | Ro-52 (+), TIF1 (+) | |  |  |  |
|  |  |  |  |  |  | |  | |  |  | |  | |  | |  | |  | |  |  |  |
| D56 | F | 54 | 155 | 58 | NO | | xerophthalmia | | Oct 2020 | Aug 2020 | | GC | | / | | / | | negative | |  |  |  |
| D57 | F | 60 | 158 | 70 | NO | | Fatty liver, hypertriglyceridemia | | Oct 2020 | Aug 2020 | | GC, HCQ, TAC | | / | | / | | Ro-52(2+) | |  |  |  |
| D58 | F | 51 | 155 | 53 | NO | | Hypertriglyceridemia, subhypothyroidism | | Oct 2020 | Dec 2019 | | GC, HCQ, MTX, CTX | | / | | / | | NXP-2 (+) | |  |  |  |
| D59 | F | 31 | 153 | 55 | NO | | NO | | Oct 2020 | Sep 2014 | | GC, HCQ, MTX | | / | | / | | no detection | |  |  |  |
| D60 | F | 38 | 150 | 35 | NO | | NO | | Oct 2020 | June 2018 | | GC, CsA | | / | | / | | MDA-5（+） | |  |  |  |
| D61 | F | 29 | 155 | 60 | NO | | NO | | Oct 2020 | Oct 2020 | | GC, MTX, CsA | | / | | / | | negative | |  |  |  |
| D62 | M | 19 | 160 | 57.5 | NO | | Fatty liver, hemolytic anemia | | Nov 2020 | Jan 2020 | | GC | | / | | / | | negative | |  |  |  |
| D63 | F | 38 | 152 | 63 | NO | | NO | | Nov 2020 | Oct 2020 | | GC, HCQ | | / | | / | | Mi-2(+++) | |  |  |  |
| D64 | F | 63 | 158 | 50 | NO | | Atrial fibrillation | | Nov 2020 | Oct 2020 | | GC | | / | | / | | negative | |  |  |  |
| **Healthy controls** | | | | | |  | |  | | |  | |  | |  | |  | |  | |  |  |
| C1 | F | 41 | 158 | 50 |  | |  | |  |  | |  | |  | |  | |  | |  |  |  |
| C2 | F | 40 | 156 | 62.5 |  | |  | |  |  | |  | |  | |  | |  | |  |  |  |
| C3 | F | 43 | 155 | 53 |  | |  | |  |  | |  | |  | |  | |  | |  |  |  |
| C4 | F | 47 | 155 | 57.5 |  | |  | |  |  | |  | |  | |  | |  | |  |  |  |
| C5 | F | 50 | 150 | 60 |  | |  | |  |  | |  | |  | |  | |  | |  |  |  |
| C6 | F | 42 | 154 | 55 |  | |  | |  |  | |  | |  | |  | |  | |  |  |  |
| C7 | F | 45 | 153 | 60 |  | |  | |  |  | |  | |  | |  | |  | |  |  |  |
| C8 | M | 47 | 165 | 65 |  | |  | |  |  | |  | |  | |  | |  | |  |  |  |
| C9 | F | 32 | 152 | 47.5 |  | |  | |  |  | |  | |  | |  | |  | |  |  |  |
| C10 | F | 65 | 160 | 60 |  | |  | |  |  | |  | |  | |  | |  | |  |  |  |
| C11 | F | 41 | 152 | 55 |  | |  | |  |  | |  | |  | |  | |  | |  |  |  |
| C12 | F | 50 | 150 | 50 |  | |  | |  |  | |  | |  | |  | |  | |  |  |  |
| C13 | M | 64 | 157 | 53 |  | |  | |  |  | |  | |  | |  | |  | |  |  |  |
| C14 | F | 47 | 156 | 55 |  | |  | |  |  | |  | |  | |  | |  | |  |  |  |
| C15 | F | 50 | 156 | 54 |  | |  | |  |  | |  | |  | |  | |  | |  |  |  |
| C16 | M | 45 | 162 | 70 |  | |  | |  |  | |  | |  | |  | |  | |  |  |  |
| C17 | M | 56 | 172 | 62.5 |  | |  | |  |  | |  | |  | |  | |  | |  |  |  |
| C18 | F | 52 | 155 | 65 |  | |  | |  |  | |  | |  | |  | |  | |  |  |  |
| C19 | F | 45 | 160 | 54 |  | |  | |  |  | |  | |  | |  | |  | |  |  |  |
| C20 | F | 47 | 155 | 52.5 |  | |  | |  |  | |  | |  | |  | |  | |  |  |  |
| C21 | F | 46 | 153 | 51 |  | |  | |  |  | |  | |  | |  | |  | |  |  |  |
| C22 | F | 55 | 150 | 59 |  | |  | |  |  | |  | |  | |  | |  | |  |  |  |
| C23 | F | 31 | 160 | 51 |  | |  | |  |  | |  | |  | |  | |  | |  |  |  |
| C24 | F | 57 | 160 | 65 |  | |  | |  |  | |  | |  | |  | |  | |  |  |  |
| C25 | F | 46 | 150 | 55 |  | |  | |  |  | |  | |  | |  | |  | |  |  |  |
| C26 | F | 54 | 151 | 60 |  | |  | |  |  | |  | |  | |  | |  | |  |  |  |
| C27 | F | 45 | 150 | 65 |  | |  | |  |  | |  | |  | |  | |  | |  |  |  |
| C28 | F | 52 | 150 | 54 |  | |  | |  |  | |  | |  | |  | |  | |  |  |  |
| C29 | F | 43 | 163 | 58 |  | |  | |  |  | |  | |  | |  | |  | |  |  |  |
| C30 | F | 54 | 148 | 49 |  | |  | |  |  | |  | |  | |  | |  | |  |  |  |

* Dermatomyositis (DM) was diagnosed according to the Bohan and Peter criteria and interstitial lung disease (ILD) was ascertained by high-resolution computed tomography. MSA: myositis-specific antibodies; GC: glucocorticoids; CTX: cyclophosphamide; CsA: cyclosporine A; MMF: mycophenolate mofetil; TAC: tacrolimus; AZA: azathioprine

Supplemental Table 2 Spearman correlations of IGFBPs with the course of disease

|  | | | **IGFBP1** | | **IGFBP2** | | **IGFBP3** | | **IGFBP4** | | **IGFBP6** | | **CTGF** | |
| --- | --- | --- | --- | --- | --- | --- | --- | --- | --- | --- | --- | --- | --- | --- |
| **Characteristics** | **Subgroup patients** | **Sample size** | **r** | **P value** | **r** | **P value** | **r** | **P value** | **r** | **P value** | **r** | **P value** | **r** | **P value** |
|  |  |  |  |  |  |  |  |  |  |  |  |  |  |  |
| **Course of DM** | All DM patients | 64 | **-0.261** | **0.037** | -0.70 | 0.585 | 0.201 | 0.112 | 0.011 | 0.932 | -0.034 | 0.788 | -0.188 | 0.136 |
|  | DM- ILD | 32 | -0.108 | 0.558 | -0.095 | 0.604 | 0.134 | 0.463 | 0.051 | 0.781 | -0.117 | 0.522 | -0.042 | 0.819 |
|  | DM-non ILD | 32 | **-0.419** | **0.017** | -0.098 | 0.593 | 0.241 | 0.184 | -0.056 | 0.762 | 0.074 | 0.688 | **-0.397** | **0.024** |
| **Course of ILD** | DM-ILD | 32 | -0.323 | 0.071 | -0.287 | 0.111 | 0.277 | 0.125 | 0.192 | 0.292 | 0.075 | 0.684 | 0.017 | 0.926 |

Bold fonts indicate statistical significance.

Supplemental Table 3 Spearman correlations of IGFBPs with pulmonary function in patients with DM-ILD

| Characteristics | Sample size | IGFBP-1 | | IGFBP-2 | | IGFBP-3 | | IGFBP-4 | | IGFBP-6 | | CTGF | | |
| --- | --- | --- | --- | --- | --- | --- | --- | --- | --- | --- | --- | --- | --- | --- |
|  |  | r | P value | r | P value | r | P value | r | P value | r | P value | r | P value |  |
| Dyspnea (mMRC scale) | 32 | 0.317 | 0.077 | **0.392** | **0.027** | -0.073 | 0.691 | 0.223 | 0.219 | 0.318 | 0.076 | **0.45** | **0.010** |  |
| HRCT | 31 | 0.264 | 0.152 | 0.036 | 0.849 | -0.166 | 0.371 | -0.129 | 0.489 | 0.205 | 0.269 | 0.191 | 0.302 |  |
| FVC (litre) | 22 | -0.289 | 0.193 | -0.249 | 0.264 | 0.309 | 0.162 | 0.411 | 0.058 | 0.006 | 0.978 | -0.047 | 0.836 |  |
| FVC (% of predicted) | 22 | -0.118 | 0.6 | -0.179 | 0.426 | 0.339 | 0.122 | **0.470** | **0.026** | 0.258 | 0.247 | -0.083 | 0.715 |  |
| FEV1 (litre) | 22 | -0.364 | 0.096 | -0.119 | 0.599 | 0.386 | 0.076 | 0.341 | 0.12 | -0.21 | 0.924 | 0.042 | 0.853 |  |
| FEV1 (% of predicted) | 22 | -0.11 | 0.625 | -0.127 | 0.573 | 0.342 | 0.119 | **0.460** | **0.033** | 0.275 | 0.215 | -0.053 | 0.816 |  |
| TLC (litre) | 22 | -0.343 | 0.118 | -0.297 | 0.179 | 0.313 | 0.155 | **0.455** | **0.034** | 0.055 | 0.807 | -0.028 | 0.903 |  |
| TLC (% of predicted) | 22 | -0.065 | 0.773 | -0.223 | 0.318 | 0.264 | 0.235 | **0.499** | **0.018** | 0.239 | 0.284 | -0.022 | 0.924 |  |
| VCmax (litre) | 21 | -0.294 | 0.197 | -0.227 | 0.322 | 0.268 | 0.241 | 0.423 | 0.056 | 0.01 | 0.964 | -0.04 | 0.862 |  |
| VCmax (% of predicted) | 21 | -0.124 | 0.592 | -0.125 | 0.59 | 0.29 | 0.202 | 0.429 | 0.052 | 0.142 | 0.54 | -0.175 | 0.449 |  |
| DLCOSB (mL/min/mmHg) | 22 | -0.402 | 0.064 | -0.115 | 0.61 | 0.216 | 0.335 | 0.103 | 0.649 | -0.272 | 0.221 | 0.001 | 0.996 |  |
| DLCOSB (of predicted) | 22 | -0.121 | 0.592 | -0.045 | 0.844 | 0.241 | 0.281 | 0.415 | 0.055 | 0.284 | 0.201 | 0.008 | 0.97 |  |
| DLCOVA (mL/min/mmHg) | 21 | -0.244 | 0.287 | -0.14 | 0.544 | 0.121 | 0.6 | -0.226 | 0.324 | -0.403 | 0.07 | -0.122 | 0.598 |  |
| DLCOVA (of predicted) | 21 | -0.07 | 0.762 | 0.15 | 0.518 | 0.081 | 0.728 | -0.098 | 0.674 | -0.081 | 0.728 | 0.202 | 0.381 |  |

Bold fonts indicate statistical significance.
